# BIDL: a brain-inspired deep learning framework for spatiotemporal processing

**DOI:** 10.3389/fnins.2023.1213720

**Published:** 2023-07-26

**Authors:** Zhenzhi Wu, Yangshu Shen, Jing Zhang, Huaju Liang, Rongzhen Zhao, Han Li, Jianping Xiong, Xiyu Zhang, Yansong Chua

**Affiliations:** ^1^Lynxi Technologies, Co. Ltd., Beijing, China; ^2^Department of Precision Instruments and Mechanology, Tsinghua University, Beijing, China; ^3^Neuromorphic Computing Laboratory, China Nanhu Academy of Electronics and Information Technology (CNAEIT), Jiaxing, Zhejiang, China; ^4^School of Automation Science and Engineering, Xi'an Jiaotong University, Xi'an, Shaanxi, China

**Keywords:** spatiotemporal processing framework, spiking neural network, global-local co-learning, synaptic plasticity, video recognition, brain-inspired computing, leaky integrate and fire, reward-modulated STDP

## Abstract

Brain-inspired deep spiking neural network (DSNN) which emulates the function of the biological brain provides an effective approach for event-stream spatiotemporal perception (STP), especially for dynamic vision sensor (DVS) signals. However, there is a lack of generalized learning frameworks that can handle various spatiotemporal modalities beyond event-stream, such as video clips and 3D imaging data. To provide a unified design flow for generalized spatiotemporal processing (STP) and to investigate the capability of lightweight STP processing via brain-inspired neural dynamics, this study introduces a training platform called brain-inspired deep learning (BIDL). This framework constructs deep neural networks, which leverage neural dynamics for processing temporal information and ensures high-accuracy spatial processing via artificial neural network layers. We conducted experiments involving various types of data, including video information processing, DVS information processing, 3D medical imaging classification, and natural language processing. These experiments demonstrate the efficiency of the proposed method. Moreover, as a research framework for researchers in the fields of neuroscience and machine learning, BIDL facilitates the exploration of different neural models and enables global-local co-learning. For easily fitting to neuromorphic chips and GPUs, the framework incorporates several optimizations, including iteration representation, state-aware computational graph, and built-in neural functions. This study presents a user-friendly and efficient DSNN builder for lightweight STP applications and has the potential to drive future advancements in bio-inspired research.

## 1. Introduction

Humans can perceive the continuously changing world, including static features such as object shapes and colors, as well as dynamic features such as motion trajectories and waveforms. These perceptions require high processing precision and need to be performed in real time with low computational power requirements. It is worth noting that brain-inspired computing features rich neural dynamics for efficiently processing temporal signals (Carlos et al., 2019) and deep learning technologies provide the capability for high-precision spatial information processing, which leads to the widespread development of the deep spiking neural networks (DSNNs) be deployed for spatiotemporal processing (Gu et al., 2019; Wu et al., 2021, 2022b). However, most of the current DSNN research focuses on dynamic vision sensor (DVS) processing or image recognition tasks. For most non-event-stream scenarios, a DSNN still lacks satisfactory accuracy. There are also many high-accuracy spatiotemporal networks, including two-stream networks (Simonyan and Zisserman, 2014a), convolutional 3D networks (Tran et al., 2015), and video transformer networks (Neimark et al., 2021) for video clip processing, networks (Han et al., 2019) for 3D imaging (image sequence) processing, long short-term memory (LSTM) (Greff et al., 2016), and transformer (Vaswani et al., 2017) networks for natural language processing (NLP) etc. The key issue is that the computational complexity and parameter size of these networks are much larger than pure spatial processing, which makes them hard for real-time high-throughput perception. Therefore, in this study, we aim to extend DSNN to lightweight generalized STP. The bio-inspired neurons are integrated into deep artificial neural networks (ANNs) and enable spatiotemporal processing with a computational complexity approaching pure spatial processing level, by introducing lightweight neural dynamics. It is revealed that temporal processing can be achieved with limited computational cost and memory footprint. We further verified that the accuracy can be improved by advanced neural models, especially for video clip processing. From this point, we develop a generalized spatiotemporal processing methodology via neural dynamics and deep neural networks (DNNs) and then designed a framework named brain-inspired deep learning (BIDL) that can adapt to a variety of modalities, such as video, DVS, text, sensor signals, etc., and finally achieves a real-time high accuracy processing. Therefore, this study extends the DSNN application domain to a much wider scope.

In another aspect, BIDL aims to provide a research platform for DSNNs. Existing frameworks mostly cater to either neuroscience researchers or machine learning researchers but not both. For computational neuroscience researchers, it is essential to have the flexibility to configure neural models, synaptic plasticity, and network structure in a research platform. Additionally, realistic applications are crucial for validating their ideas. From the perspective of neural simulators, there are already several simulators available, ranging from high-accuracy simulation (Carnevale and Hines, 2006) to large-scale simulation (Gewaltig and Diesmann, 2007). Some frameworks also accelerate network simulation using GPUs (Yavuz et al., 2016; Golosio et al., 2021). In terms of applications, Bekolay et al. (2014) and Wang et al. (2022) provided rich examples of bio-inspired networks and dynamics. BIDL offers similar design flexibility to these frameworks and enables vectorized acceleration for neuron populations and synapse connections using PyTorch. Furthermore, BIDL enables back propagation through time (BPTT) training for advanced neural models.

Machine learning researchers require a fast development platform for DSNNs with a deep neural network (DNN) design style. Support for global-local learning is essential for validating brain-inspired local learning with gradient-based global learning. Among the frameworks that fulfill these requirements, some frameworks such as Hazan et al. (2018), Rasmussen (2019), and Bohte et al. (2000) enable spiking neural network (SNN) designs using deep learning frameworks such as Jax, PyTorch, and TensorFlow. Among them, Fang et al. (2020) enabled BPTT learning, which achieves high accuracy. Inspired by these frameworks, BIDL provides a rapid development platform for designing and training DSNNs using a DNN-style approach. BIDL also introduces a configuration file that integrates networks with datasets and pre-processing pipelines, similar to OpenMMLab (MMCV-Contributors, 2018), enabling efficient DSNN design. Additionally, BIDL introduces a unified flow for global-local co-learning in DSNNs, including BPTT learning and programmable generalized synaptic plasticity. Therefore, BIDL serves as a unified research framework for both neuroscience and machine learning researchers.

Moreover, BIDL incorporates a range of optimizations to fit the designed network to neuromorphic chips, particularly focusing on the iteration representation. Since most neuromorphic chips operate in a timestep-driven manner, some frameworks are specifically designed for such cases (Gewaltig and Diesmann, 2007; Davison et al., 2009; Wang et al., 2022). However, for speeding up GPU processing, an iteration within each temporal layer proves to be a better choice, as applied in SpikingJelly (Fang et al., 2020). Consequently, BIDL supports both internal and external iteration methods, allowing training within a single framework while utilizing the same design flow.

The main contributions of this study are as follows:

(1). BIDL provides a unified DSNN design flow for a range of STP applications, including video clip processing, moving trajectory processing, dynamic vision sensor (DVS) recognition, 3D medical imaging classification, and NLP task. These experiments demonstrate the efficiency of BIDL, achieving high accuracy while consuming significantly less computation than traditional convolutional 3D (Conv3D) or LSTM approaches. Finally, real-time processing of these experiments on embedded platform is realized.

(2). As a research framework for neuroscience and machine learning researchers, BIDL facilitates the exploration of various differentiable neural models such as the leaky integrate-and-fire (LIF) and LIF+ models. It also supports different neural configurations, including analog spike and residual membrane potential (RMP). Furthermore, BIDL enables global-local co-learning through the use of back-propagation through Time (BPTT) for global learning and generalized synaptic plasticity rules for local learning. To demonstrate the exploration capability of BIDL, we provide an anti-noise example utilizing BPTT and localized plasticity co-learning as well as a DVS recognition example employing various LIF+ neurons.

(3). To ensure compatibility with neuromorphic chips and GPUs, the BIDL framework incorporates both internal iteration and external iteration of timesteps into a unified design flow. Additionally, we propose a state-variable indicated computational graph as a representation of the networks, which facilitates seamless integration with downstream SNN compilers.

The article is organized as follows: Section 2 illustrates the generalized spatiotemporal processing network through neural dynamics. Section 3 discusses the characteristics of the BIDL research platform, catering to the needs of researchers in the field. Section 4 presents optimizations and considerations for deploying BIDL on neuromorphic chips. Section 5 provides diverse examples of spatiotemporal processing, advanced neural models, and global-local co-learning. Section 6 presents a discussion on the design choices of the BIDL framework. Finally, Section 7 concludes the study.

## 2. Generalized spatiotemporal processing via neural dynamics

Generalized lightweight spatiotemporal processing requires several criteria. First, it needs to be a generalized method that can adapt to a variety of modalities, such as video, DVS, text, and sensor signals. Second, it should have high accuracy in processing both spatial and temporal information. Third, it should be lightweight in terms of computation and memory usage, enabling real-time low-latency processing. In this section, we will discuss how BIDL meets these requirements.

For a generalized spatiotemporal framework, the neural networks in BIDL treat all spatiotemporal source signals as spatiotemporal tensors. These tensors contain both spatial and temporal information, forming a spatio-temporal (ST) tensor with the shape [*B, T, C, H, W*]. In this study, *B* represents the batch size, *T* denotes the total timesteps, *H* and *W* represent height and width, respectively, and *C* denotes the number of channels. This spatiotemporal tensor format allows the representation of spatial information in [*C, H, W*] and temporal information with *T* timesteps.

**Figure 1:**
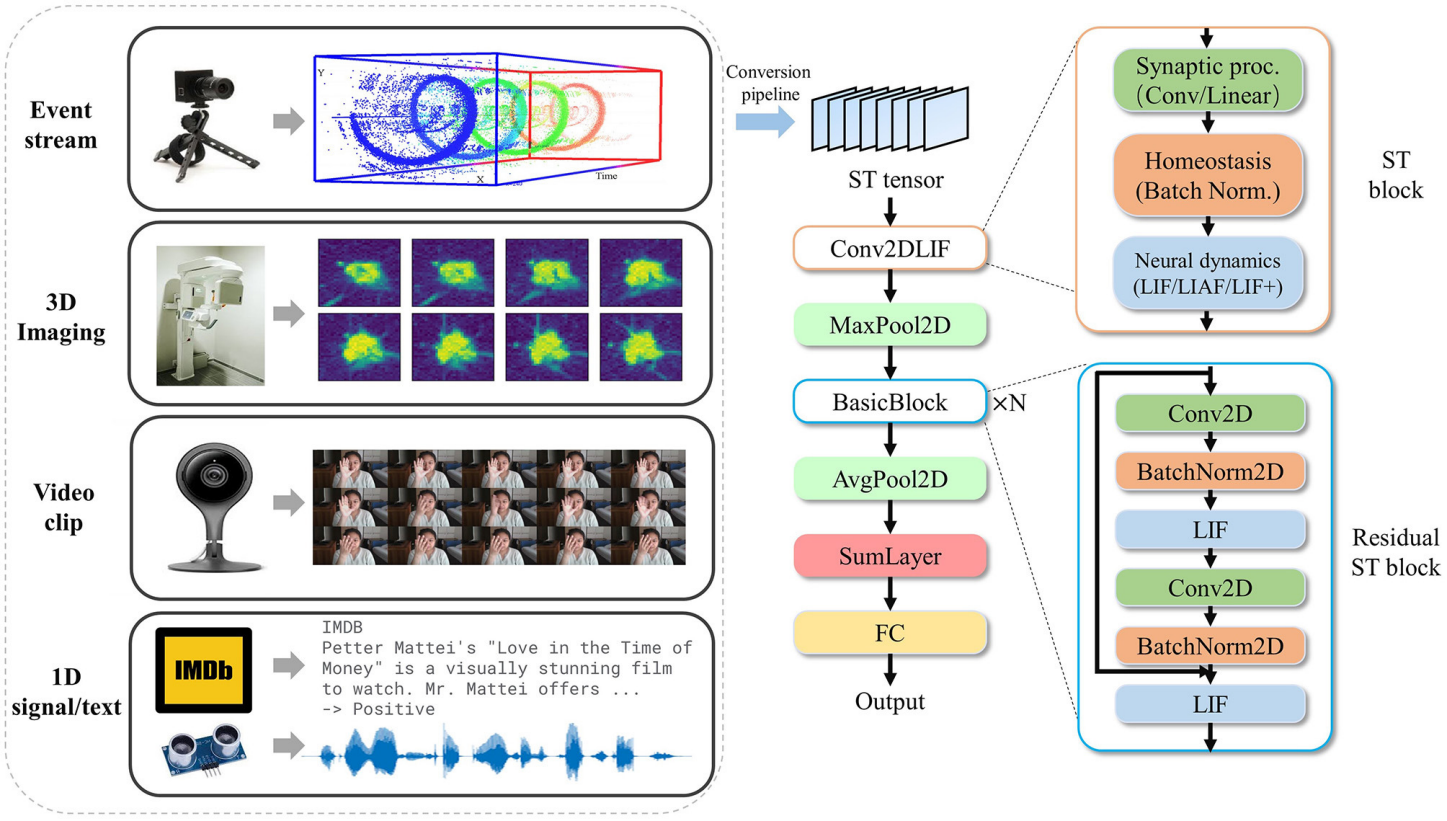
Proposed ST processing on various input modalities, including event-stream, video clip, 3D imaging, and 1D signals (including text). The data are first converted to an ST tensor via a conversion pipeline and then processed by a DSNN with ST blocks. The ST block consists of synaptic processing, such as convolution or linear layers, homeostasis, such as batch normalization, and neural dynamics, including LIF/LIAF/LIF+. The event-stream demonstration sub-figure is modified from Hinz et al. (2017).

BIDL requires a data pre-processing procedure to convert the source data into the ST tensor format, as illustrated in Figure 1. For a video clip, the temporal tensor represents a sequence of frames. The video frames undergo a sampling rate conversion followed by image preprocessing to derive the temporal tensor. For DVS signals, the event format represented as (*x, y, t, ps*) over a time duration *t* ∈ *t*_*s*_ is collected and forms a frame. In this study, *x* and *y* denote the pixel location, and two channels represent the increase and decrease of light intensity, respectively. Each pixel is represented by an integer value indicating the count of events at that location. To satisfy the spiking format for SNN, it can also be transformed into a binary format, where one or several neurons represent a pixel. For 3D imaging data, each slice of the 3D imaging can be treated as a “frame,” allowing the 3D data direct transformation into a temporal tensor. For purely temporal signals, such as text or voice, they can be represented in the 5D format with *H* = 1 and *W* = 1. However, for easier understanding in most cases, we use a 3D tensor format with the shape [*B, T, S*], where *S* represents the dimension of hidden states. Therefore, multiple modalities can be converted into a unified ST tensor format for neural network processing. BIDL also provides a set of data pre-processing pipelines for converting data formats into the unified ST format. It is important to note that this procedure is defined by the user, and the methods for processing new data sources may vary. Detailed information about the pre-processing for each experiment can be found in Section 5.

**Figure 2:**
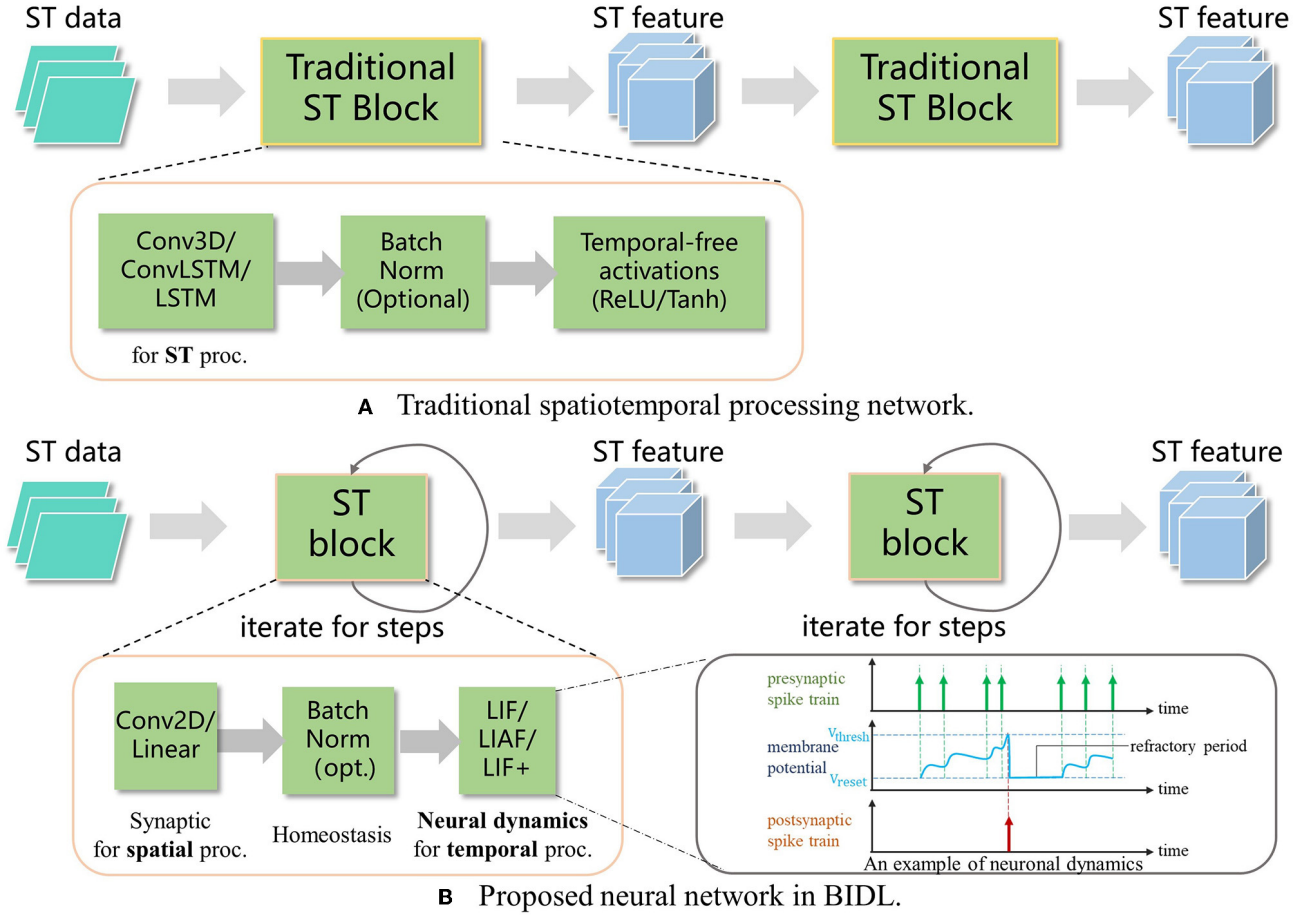
Proposed spatiotemporal processing by utilizing neural dynamics for temporal processing. **(A)** Traditional spatiotemporal processing via 3D convolution or Convolutional LSTM. **(B)** The proposed ST block (such as ConvLIF/ConvLIAF) is applied for spatiotemporal processing, where 2D convolution or linear operations are employed for spatial processing, and bio-inspired neurons with dynamics are introduced for temporal processing. Due to the lower computational requirements of 2D convolution and bio-inspired neurons compared to Conv3D or ConvLSTM, the network becomes lightweight and enables real-time processing.

**Figure 3:**
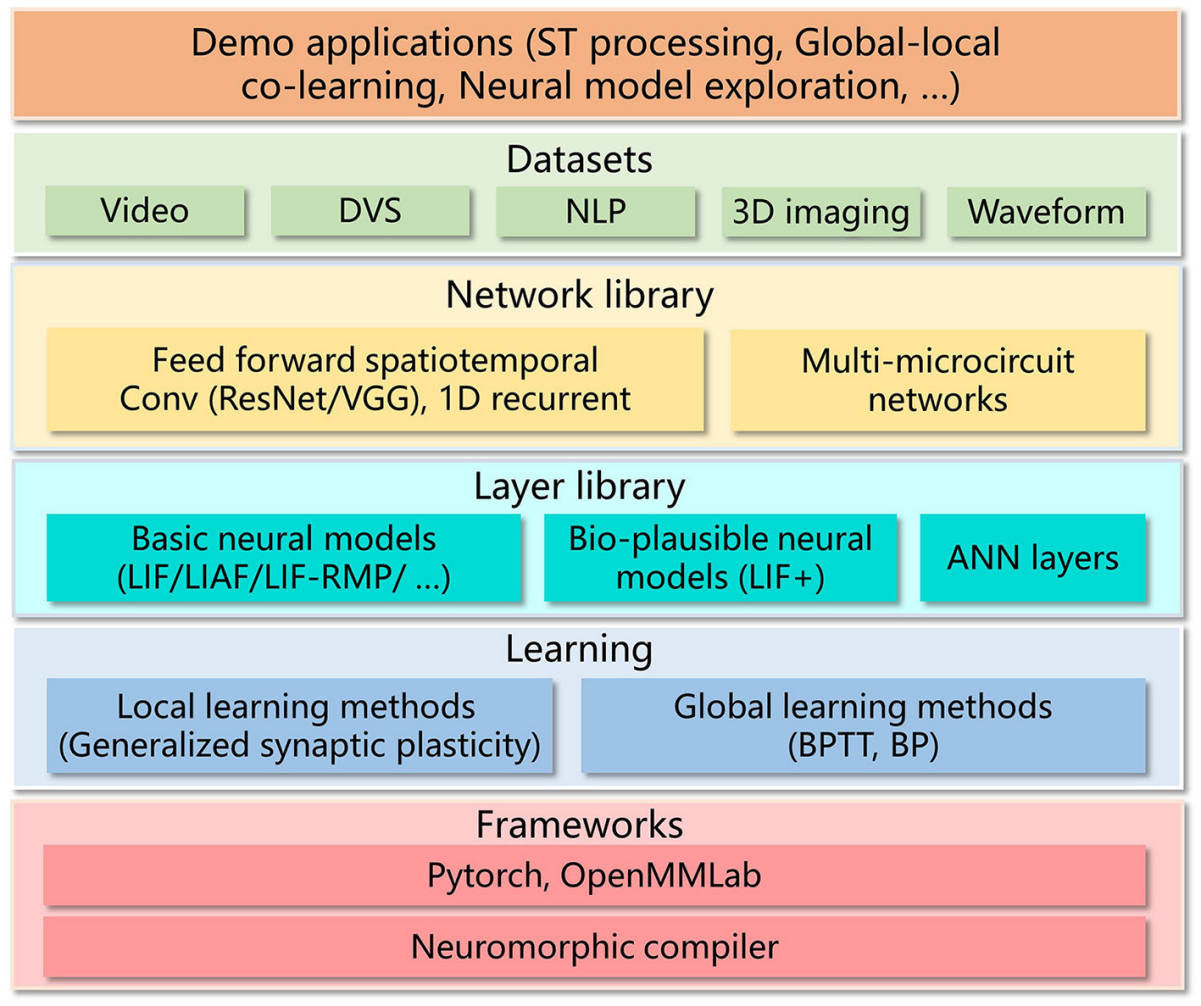
Top-level architecture of BIDL. BIDL is based on PyTorch and OpenMMLab (MMCV-Contributors, 2018). It consists of learning modules, a layer library (neural models), a network library, datasets, and demo applications. The networks designed by BIDL can be ported to the neuromorphic compiler via a state-aware computational graph representation.

For high-accuracy spatiotemporal processing, BIDL utilizes a DSNN, where ANN layers and SNN layers are interleaved and stacked in a network. With the advantages of deep learning technologies, ANN layers, including convolution, batch normalization, pooling, dropout, linear, residual connections, and attention mechanisms, are proficient in spatial (image) processing, as shown in Figure 1. Additionally, the backbone network of DSNN can be directly adopted from DNN, such as ResNet (He et al., 2016) or VGG (Simonyan and Zisserman, 2014b). On the contrary, SNN layers, such as LIF, can be inserted into these DNNs to introduce temporal processing capability. Therefore, ANN layers are responsible for spatial processing, while SNN layers handle temporal processing. It has been demonstrated that the neural dynamics of these neural models can effectively extract temporal information (Wu et al., 2021, 2022a). In particular, the ConvLIF is an ST block that incorporates convolution, batch normalization, and LIF, making it suitable for lightweight spatiotemporal processing. The ConvLIAF block is an improved ST block that replaces spike activations with analog activations while maintaining the neural dynamics of LIF (Wu et al., 2021), thereby enhancing spatial signal transfer accuracy. These ConvLIF/ConvLIAF blocks can be considered as the fundamental building blocks for constructing spatiotemporal networks, as shown in Figure 2. We can also leverage basic backbone networks from DNN designs and insert neural models to enable temporal processing, such as ResNet-LIF or VGG-LIAF. These networks can be trained in BIDL using global learning methods, such as BPTT, or learned through local learning methods, such as Hebb or spike-timing-dependent plasticity (STDP). BPTT provides high-accuracy processing through supervised learning, while local methods offer unsupervised or weakly supervised learning, which can be used for adapting to new tasks or environments. The top-level architecture of the proposed framework is illustrated in Figure 3.

### 2.1. Definition of the LIF/LIAF neural model

The definition of LIF, Leaky Integrate and Analog Fire (LIAF), and Residual Membrane Potential (RMP) applied in this study has been previously illustrated in Han et al. (2020), Wu et al. (2021), and Wu et al. (2022b). For an easy understanding of the proposed framework, we reintroduced the definitions as follows.

The original LIF model is described in a differential function (Ferré et al., 2018; Roy et al., 2019) to reveal the neural dynamics, following the equation


(1) $$\begin{array}{l} \tau \frac{dV_{j}\left(t\right)}{dt}=-\left(V_{j}\left(t\right)-V_{rest}\right)+RI_{j}\left(t\right), \end{array}$$

where *j* represents the neuron index. τ is the timing factor of the neuron, *V*_*rest*_ is the resting potential, and *I*_*j*_(*t*) is the input current. When *V*_*j*_(*t*) reaches a certain threshold *V*_*th*_, a spike is emitted, and the *V*_*j*_(*t*) is reset to an initial value *V*_*reset*_.

We introduce the Euler method (Wu et al., 2018; Neftci et al., 2019) on Eq. 1 to obtain an iterative representation in discrete-time for easy inference and training. We define Δ*t* as the sampling duration which is a small fraction of time, with the Euler method, the equation can be solved numerically:


(2) $$\begin{array}{l} V_{j}\left(t\right)=V_{j}\left(t-\text{Δ}t\right)+\frac{\text{Δ}t}{\tau }\left(-V_{j}\left(t-\text{Δ}t\right)+V_{rest}+RI_{j}\left(t-\text{Δ}t\right)\right). \end{array}$$

After sampling with a sampling rate of 1/Δ*t*, we denote the timestep as *t*_*n*_, where *t* = *t*_*n*_Δ*t*, then we have


(3) $$\begin{array}{l} V_{j}\left(t_{n}\text{Δ}t\right)=V_{j}\left(\left(t_{n}-1\right)\text{Δ}t\right)\left(1-\frac{\text{Δ}t}{\tau }\right)+\frac{\text{Δ}t}{\tau }V_{rest}+\frac{\text{Δ}t}{\tau }RI_{j}\left(\left(t_{n}-1\right)\text{Δ}t\right). \end{array}$$

For simplicity, we further define $\alpha =1-\frac{\text{Δ}t}{\tau }$, $\beta =\frac{\text{Δ}t}{\tau }V_{rest}$, and $r=\frac{R}{\alpha }\frac{\text{Δ}t}{\tau }$, then Eq. 3 can be written as


(4) $$\begin{array}{l} V_{j}\left(t_{n}\text{Δ}t\right)=\alpha \left(V_{j}\left(\left(t_{n}-1\right)\text{Δ}t\right)+rI_{j}\left(\left(t_{n}-1\right)\text{Δ}t\right)\right)+\beta \end{array}$$

In the discrete form, we skip the notation Δ*t*, therefore, we get


(5) $$\begin{array}{l} V_{j}^{t_{n}}=\alpha \left(V_{j}^{t_{n}-1}+rI_{j}^{t_{n}-1}\right)+\beta . \end{array}$$

Therefore, we obtained the LIF representation in a discrete form. In the remaining part of this article, all the expressions are in a discrete form, and we use *t* instead of *t*_*n*_ to represent the timestep for simplicity.

We further pack a group of neurons in a tensor for a more compact expression, where *I*^*t*^, *V*^*t*^, *V*_*th*_, *V*_*reset*_, α, β, and *r* are all in a tensor format, and we have re-written LIF/LIAF in following calculation procedure:

(a). Accumulate input current with the previous membrane potential:


(6) $$\begin{array}{l} V_{m}^{t}=V^{t-1}+r\cdot I^{t}, \end{array}$$

where *V*^*t*−1^ and *V*^*t*^ refer to the previous and current membrane potential, respectively. The input current usually comes from convolutional synaptic calculation or linear (fully connected) synaptic calculation.

(b). Compare with the threshold and fire:


(7) $$\begin{array}{l} F^{t}=V_{m}^{t}\geq V_{th}, \end{array}$$

where *F*^*t*^ is the fire signal. For each $F_{j}^{t}$ in *F*^*t*^, $F_{j}^{t}=1$ indicates a firing event; otherwise, $F_{j}^{t}=0$.

(c). Reset the membrane potential when fired:


(8) $$\begin{array}{l} R^{t}=F^{t}\cdot V_{reset}+\left(1-F^{t}\right)\cdot V_{m}^{t} \end{array}$$

When using residual membrane potential (RMP) (Han et al., 2020), a soft reset instead of a hard reset is applied:


(9) $$\begin{array}{l} R^{t}=F^{t}\cdot \left(V_{m}^{t}-V_{th}\right)+\left(1-F^{t}\right)\cdot V_{m}^{t} \end{array}$$

(d). Perform leakage:


(10) $$\begin{array}{l} V^{t}=\alpha \cdot R^{t}+\beta \end{array}$$

where α and β represent the multiplicative decay and additive decay, respectively.

(e). Output:


(11) $$Y^{t}=\{\begin{array}{ll} F^{t}, & \text{for LIF}\\ f(V^{t}), & \text{for LIAF,} \end{array}$$

where *f*(*x*) is the analog activation function.

In the following illustrations, we term convolutional integration with LIAF/LIF as ConvLIAF/ConvLIF, respectively. We use an RMP flag (such as ConvLIF-RMP) when a soft reset is used. In addition, parameters *V*_*th*_, *V*_*reset*_, α, and β may vary for each convolutional channel termed as channel sharing mode (CSM) or be the same for all neurons termed as all sharing mode (ASM). Since *r* is a resistance to the input current, and the input current is derived from synaptic integration, we discarded it since it can be represented as a gain factor of the synaptic weights. There are also many variations for the proposed LIF, which are noted as LIF+. Please refer to Section 3.1.1 for details.

### 2.2. Lightweight processing

To verify the lightweight characteristics of the proposed networks in BIDL, we summarized the computational cost and memory cost of the proposed ST block, comparing it with traditional ST blocks such as Conv3D and ConvLSTM. The comparison is shown in Table 1.

**Table 1:** Formulas for calculating the computational complexity and the weights of different spatiotemporal layers.

**Layer**	**MUL**	**ADD**	**Weights**
ConvLIAF	(*Q*+1)·*R*	(*Q*+2)·*R*	(*Q*+1)·*C*
ConvLIF	*R*	(*Q*+2)·*R*	(*Q*+1)·*C*
Conv2D (TD)	*Q*·*R*	*Q*·*R*	(*Q*+1)·*C*
Conv3D	*U*·*Q*·*R*	*U*·*Q*·*R*	(*U*·*Q*+1)·*C*
ConvLSTM	(4·(*Q*+*I*·*J*·*C*)+3)·*R*	(4·(*Q*+*I*·*J*·*C*)+1)·*R*	(*Q*+*I*·*J*·*C*+1)·4·*C*

TD refers to the time-distributed operation, i.e., duplicate over time.

**Figure 4:**
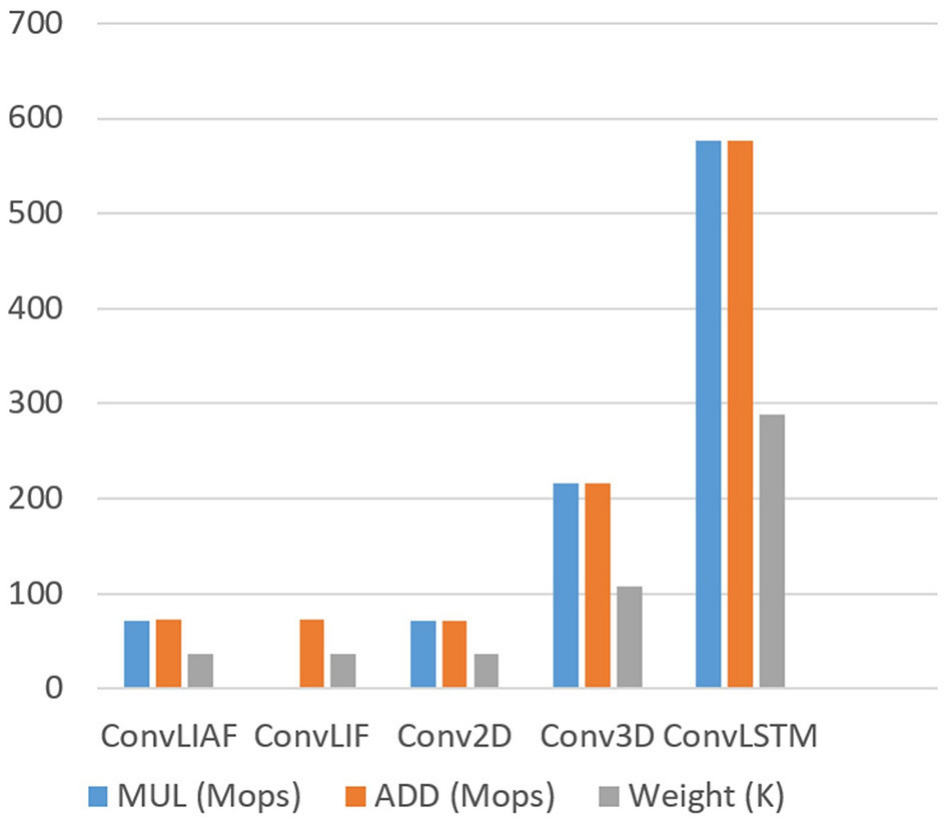
Computational complexity and number of weights of a representative ST block for an example parameter setting (T = 8, H = W = 16, C = K = 64, U = I = J = 3). It reveals that ConvLIF/ConvLIAF consumes similar computational resources and parameters to (time-distributed) Conv2D, while much fewer than Conv3D and ConvLSTM.

We assume that the hidden state and output have the same spatiotemporal tensor size, denoted as [*B, T, H, W, C*], where *B* = 1. (*I, J*) represents the convolution kernel size of Conv2D, ConvLIF, ConvLIAF, and ConvLSTM, while (*U, I, J*) represents the convolution kernel size of Conv3D. The variable *K* denotes the size of input channels. For the sake of comparison, in Table 1, we use *R* and *Q* to represent *T*·*H*·*W*·*C* and *I*·*J*·*K*, respectively. We also fix these parameters to a set of typical values and plot the corresponding values of computational operations and weight parameters, as shown in Figure 4.

The results from Table 1 and Figure 4 reveal that the computational overhead of ConvLIAF is not significantly different from that of Conv2D (time-distributed). Furthermore, ConvLIF consumes fewer multiplications thanks to its spiking format. It can be observed that, for the same temporal tensor shape, both ConvLIAF and ConvLIF achieve a computational reduction of 3x compared to Conv3D and 8x compared to ConvLSTM, as shown in Figure 4. Therefore, compared with Conv3D and ConvLSTM, ConvLIAF can save a significant amount of computational resources and storage overhead. The impact on accuracy is reported in literature Shi et al. (2020) and Wu et al. (2021), and is also discussed in our experiment section.

## 3. BIDL: an easy-to-use platform for SNN researchers

The BIDL platform is designed to cater to two main types of researchers: computational neuroscience researchers and machine learning researchers.

### 3.1. For computational neuroscience researchers

Computational neuroscience researchers primarily focus on building various neural models, exploring synaptic plasticity rules, and studying network structures. Hence, BIDL provides support for these features, and spatiotemporal applications serve as experimental examples to support their theoretical viewpoints.

#### 3.1.1. Neural model support

**Figure 5:**
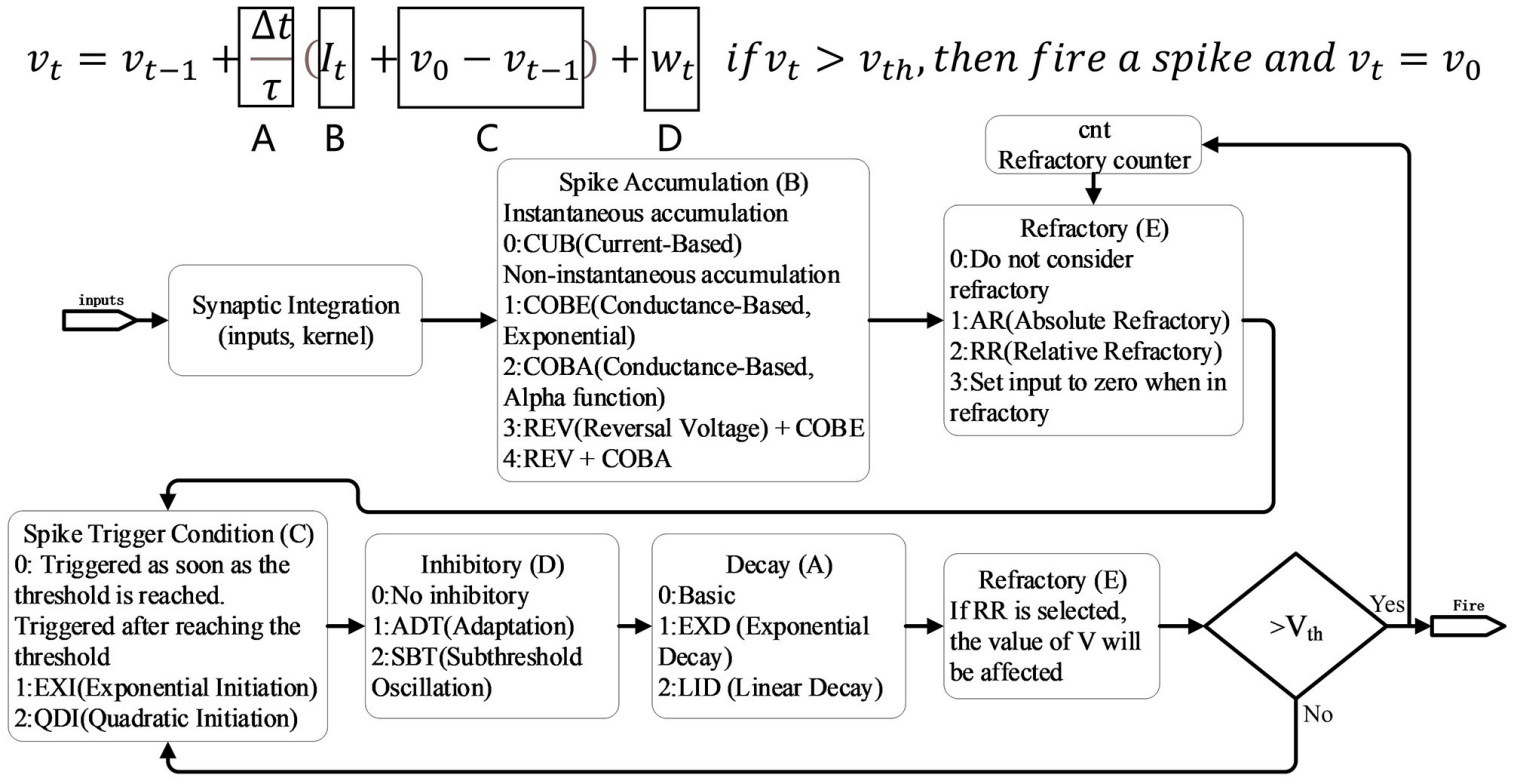
LIF+ neuron with configurable modes. Each mode can be treated as an improvement of the original simplified LIF model, including A: decay, B: spike accumulation, C: spike trigger condition, D: inhibitory, and E: refractory. Detailed definitions can be found in Lee et al. (2018).

Unlike most computational neuroscience frameworks such as Nest (Gewaltig and Diesmann, 2007) and Neuron (Carnevale and Hines, 2006), where neurons cannot be directly trained using gradient-based learning methods, BIDL introduces a group of neurons called LIF+ that can be trained directly using backpropagation through time (BPTT) with surrogate gradients. The LIF+ neurons are derived from the original leaky integrate and fire (LIF) model by incorporating improvements in key aspects of the differential equations to enhance neurodynamics, as inspired by Lee et al. (2018). Figure 5 illustrates the decomposition of LIF+ into five stages, each offering several choices for improving the similarity to biological neural dynamics. Detailed definitions of each mode can be found in Lee et al. (2018). Additionally, BIDL supports customized neural models, allowing users to define their own models expressed as sub-networks in PyTorch.

#### 3.1.2. Generalized synaptic plasticity rules

**Figure 6:**
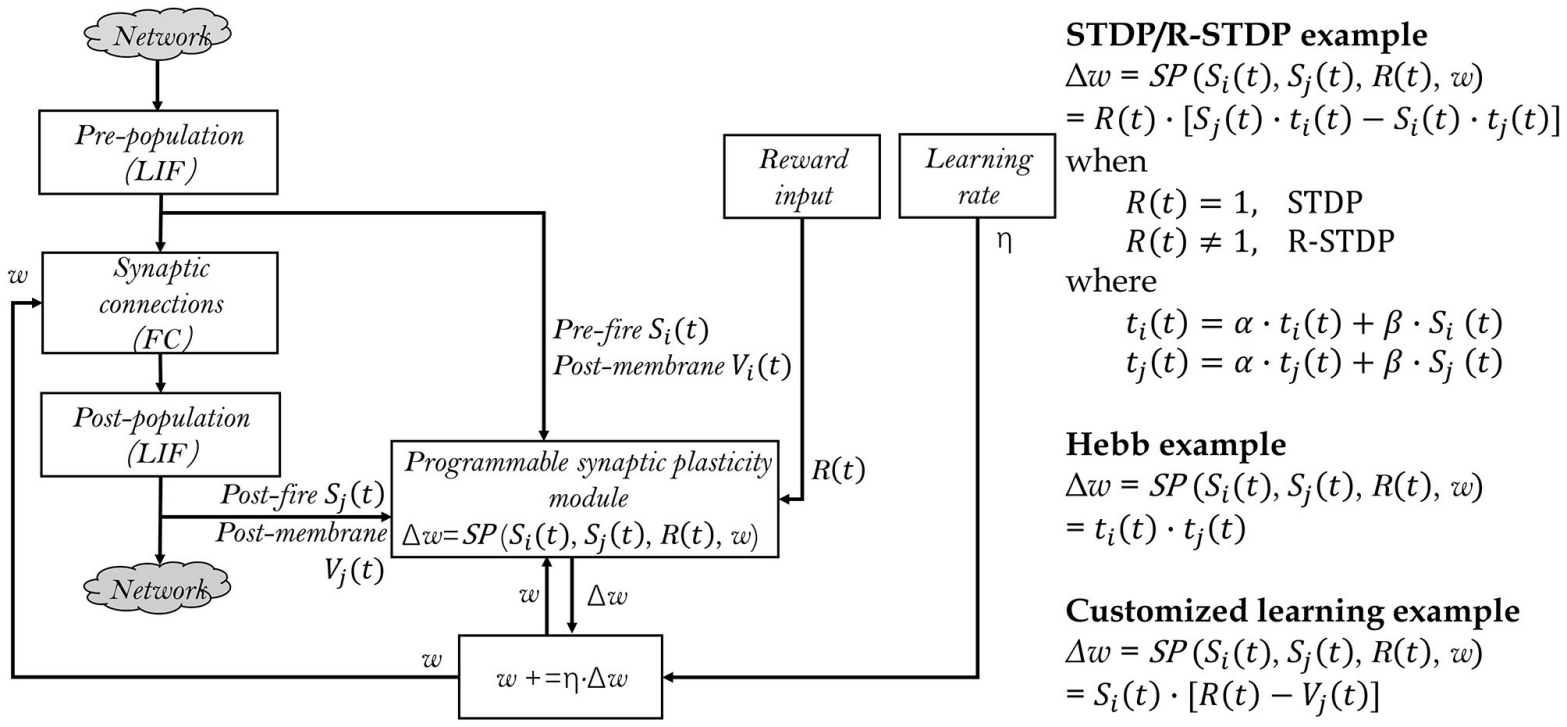
Generalized local learning module, which can be configured to support STDP, R-STDP, Hebb, or user-defined synaptic plasticity rules. The two LIF populations and synaptic connections are part of the neural network, while the remaining parts of the neural network are denoted as “Network” for simplicity. This also indicates that only a subset of the synaptic weights of interest are adjusted during local learning.

Exploring various synaptic plasticity approaches is common among computational neuroscience researchers. In BIDL, we introduce a local learning module, which is a customizable programmable module that supports Hebb, spike-timing dependent plasticity (STDP), and reward-modulated STDP (R-STDP), as shown in Figure 6. This module receives spikes and optionally membrane potentials from the current neuron population (e.g., a LIF layer) as well as the previous neuron population. It also receives the reward signal from the external environment and reads the previous weights of the synaptic array. Using the user-defined update function *SP*, the module calculates the weight update value Δw.

### 3.2. For machine learning researchers

#### 3.2.1. A deep learning style SNN builder

For machine learning researchers, their focus is on how SNNs can assist DNNs in efficient spatiotemporal processing and designing SNNs using a DNN building approach. BIDL leverages popular DNN designing frameworks such as PyTorch and OpenMMLab (MMCV-Contributors, 2018) to provide a familiar development environment. Researchers can reuse DNN backbones such as ResNet and VGG for building SNNs in BIDL. The networks can be trained using backpropagation through time (BPTT) similar to pure DNNs, enabling DNN researchers to seamlessly transition to SNN work with minimal adaptation required.

#### 3.2.2. Global-local learning support

Global learning methods such as BPTT excel in learning from supervised information and achieve high accuracy for many applications. However, they come with a high computational burden and memory usage since the membrane potentials and activations of each timestep in the forward propagation need to be recorded for the backward propagation. On the contrary, local learning methods based on synaptic plasticity rules offer better computational energy efficiency but often suffer from lower accuracy. In this regard, we propose a hybrid training approach that integrates global-local learning into a unified flow, allowing interleaved operation of global learning and local learning.

During global learning, the synaptic plasticity learning module is detached, and the network is trained using BPTT. During local learning, the weights are adjusted via local plasticity, and BPTT is not required. This hybrid approach is particularly useful in pre-training-finetuning scenarios, where the network is first trained globally using BPTT and then fine-tuned using reward-modulated STDP (R-STDP) to adapt to changes in the environment.

## 4. Mapping optimizations for neuromorphic chips

**Figure 7:**
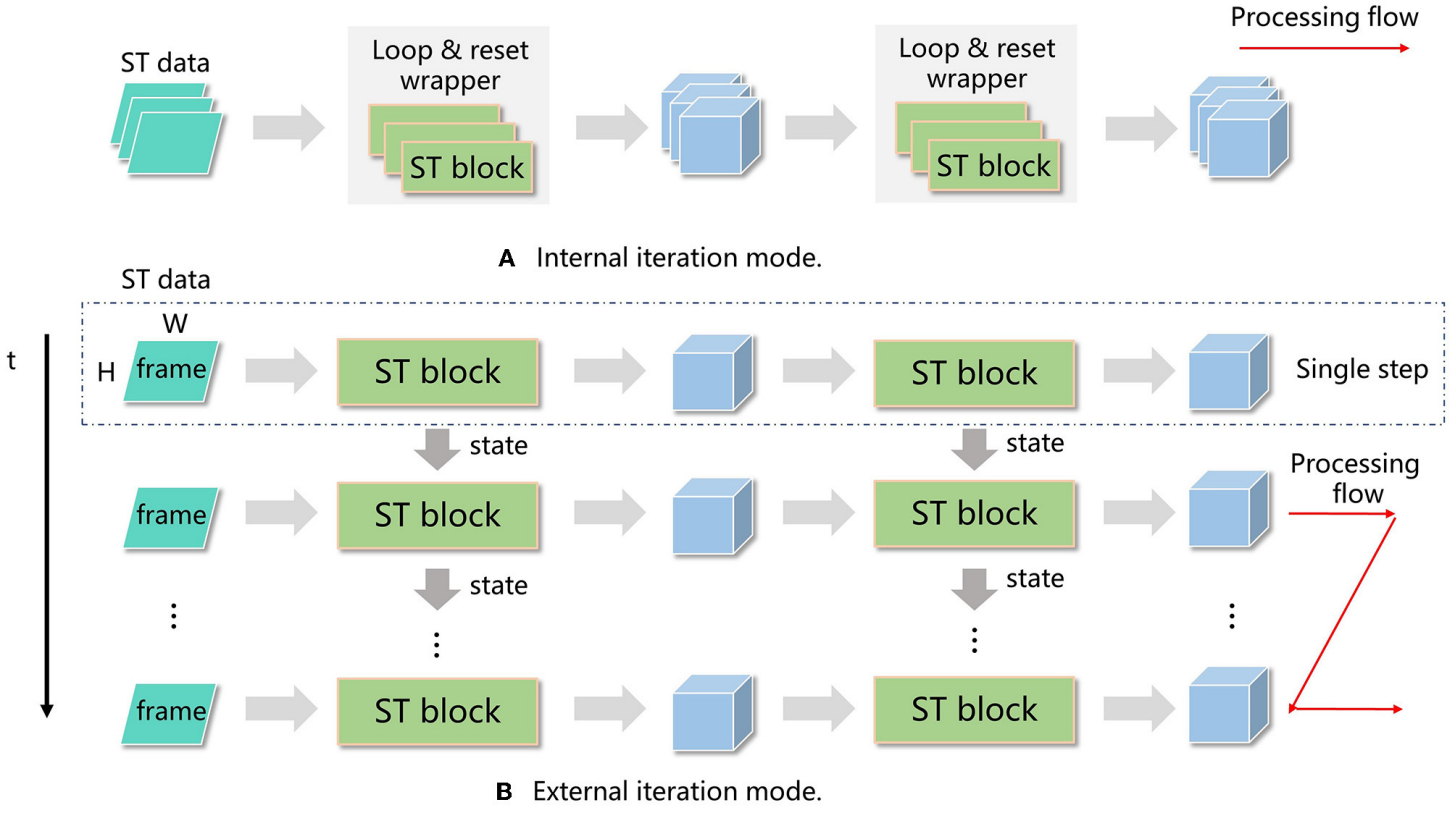
Processing flow of internal iteration (IIM) **(A)** and external iteration (EIM) **(B)**. In IIM, a loop and reset wrapper is introduced in each spatiotemporal (ST) block, and all the timesteps for the current block are calculated before moving to the next block. In EIM, the timestep iteration is located outside the network, and all the layers in the network are processed before moving to the next timestep.

BIDL can also serve as an application builder for neuromorphic chips, taking into consideration their unique characteristics and constraints. It generates a computational graph that can be used by subsequent neuromorphic compiling tools. Currently, neuromorphic chips accept computational descriptions with specific constraints, including being timestep driven, caching state variables (membranes) for use in the next timestep, and parameter quantization for memory savings.

(1). Most neuromorphic chips operate in a timestep driven manner, where a timestep iteration is located outside the neural network. The device computes all the layers in each timestep and then switches to the next timestep. In contrast, GPU-based SNN frameworks usually locate the timestep iteration within each layer, resulting in outputs with an extra temporal dimension. To address this difference, we designed two modes of operation: internal iteration mode (IIM) and external iteration mode (EIM), shown in Figure 7.

In IIM, single-step layers such as convolutions and LIF neurons are wrapped by a timestep iteration and a reset phase, forming iterated layers. The network is built based on these iterated layers, which have a temporal dimension on both the input and output. In EIM, the single-step network is built directly using single-step layers, and the network is invoked *T* times, where *T* is the number of timesteps. Both modes can be trained using BPTT and achieve similar accuracy.

IIM offers more flexibility as each iterated layer can have its own total number of timesteps, and temporal transforms (such as decreasing the number of timesteps) or attention operations can be applied between timesteps for temporal information aggregation.

For classification tasks, where there is a single timestep at the end of the network, a temporal aggregation layer (e.g., sum or average) is applied before the classification head to reduce the number of timesteps to 1. This is straightforward in IIM, but for EIM, all layers share the same timestep setting, making direct temporal aggregation impractical. In such cases, we use an accumulator instead of temporal aggregation. Mathematically, instead of calculating the output as the sum of inputs over timesteps, i.e., $Out=\overset{T-1}{\underset{i=0}{\sum }}Input\left(i\right)$, we use an accumulator *Acc*(*i*) defined as *Acc*(*i*) = *Acc*(*i*−1)+*Input*(*i*), with a reset value of *Acc*(−1) = 0, and the output is obtained as *Out* = *Acc*(*T*−1).

(2). When compiling the network for neuromorphic chips, we utilize EIM, and only a single-iteration network is compiled. We represent the network in a computational graph format, where each node corresponds to a functional layer, and each edge represents an intermediate tensor. In a traditional computational graph, these tensors can be destroyed after the graph computation is finished, meaning all the variables survive only at the current timestep. However, some state tensors, such as membrane potentials, need to be carried over to the next timestep and updated in place. To handle this, we introduce two additional nodes to the graph: the load node and the save node. The load node can be associated with a constant tensor node, which provides the initial value during the initialization stage or is reset by the user. Resetting is typically done when calculating a new sample and requires erasing the membrane potential from the previous sample. To distinguish between different state tensors in the graph, we assign them unique string identifiers, generated as universally unique identifiers (UUIDs). The load and save nodes are represented as customized layers in PyTorch.

(3). The compiled network is executed on the neuromorphic chip using a runtime tool. Within a single input sample, the runtime tool iterates through each timestep, provides input data to the device, performs the network inference, and obtains the output. The input data consists of a sequence of frames, with one frame processed at each timestep. For networks that generate single-step outputs (e.g., classification or detection), only the output of the last timestep is required. After executing all the timesteps, a state reset command is issued to reset the membrane potential, preparing for the next sample.

(4). Using PyTorch layers to represent the computational process of neurons may introduce additional nodes to the graph, making it more complex and less recognizable for optimization on neuromorphic chips. In most devices, basic neural models such as LIF neurons have dedicated circuits for implementation. Therefore, we rewrote the LIF neuron in PyTorch using two customized layers specifically for inference (not for training). These layers, cmp_and_fire and reset_with_decay, represent the compare-to-threshold and firing process and the membrane reset and decay process, respectively. These blocks can be treated as black boxes in the computational graph and recognized directly by neuromorphic chips, enabling circuit-level optimization.

**Figure 8:**
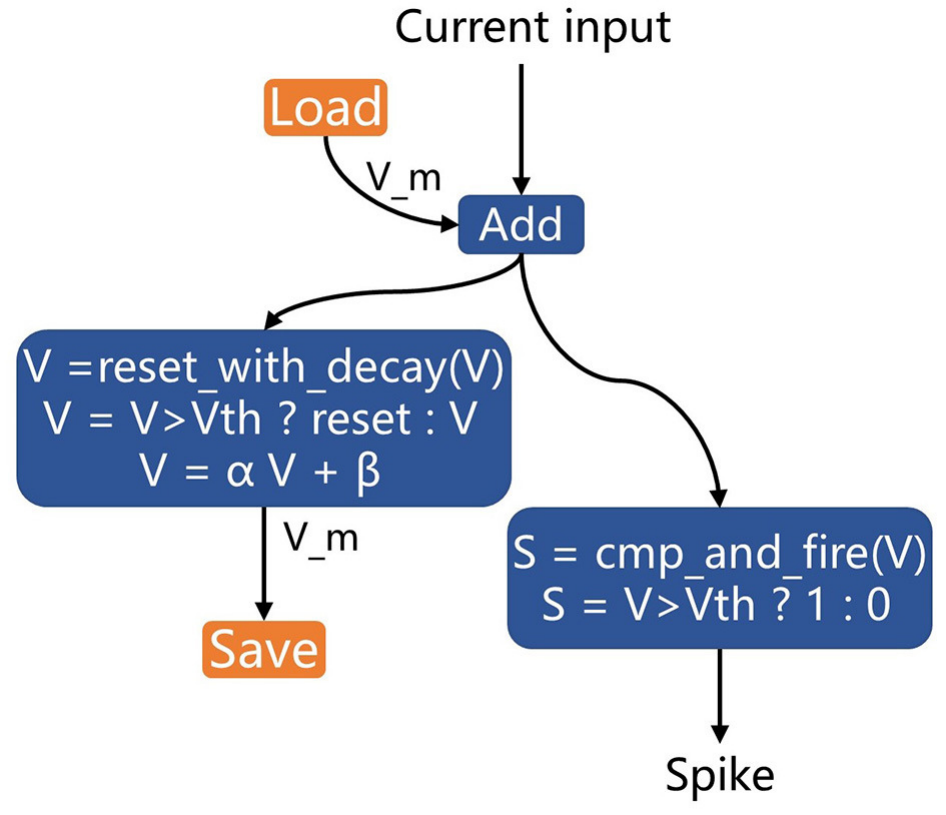
LIF neuron implementation with hardware-defined built-in blocks for neuromorphic chip acceleration.

Figure 8 illustrates the implementation of LIF neurons using the hardware-defined built-in blocks for acceleration on neuromorphic chips. The previous membrane potential is loaded and then added to the input post-synaptic current. The cmp_and_fire block is used to calculate the spike, and the reset_with_decay block is applied to update the membrane potential and perform decay.

(5). To reduce memory footprint and computational energy, quantization is applied. BIDL provides float16 training, which utilizes the float16 support in PyTorch with the loss amplified by a user-defined setting, such as 512. These mechanisms discussed in this sub-section have been deployed in the brain-inspired chip Lynchip KA200.^1^

## 5. Experiments

In this section, we demonstrate several spatiotemporal applications in BIDL across multiple modalities, including video, DVS, 3D imaging, and NLP. We also evaluate several models in LIF+ with various neuronal variations to illustrate the neural modeling capabilities. Finally, we showcase local-globalized co-learning for high-accuracy transfer learning.

### 5.1. Applications

#### 5.1.1. Video processing

**Video gesture recognition (Jester):** Currently, there is limited literature discussing video processing with spiking neural networks, and most existing networks for video processing have high computational complexity, consuming more power than image processing. In this study, we propose an SNN approach, ResNet18-LIF, which achieves a similar computational cost as the traditional ResNet-18, to demonstrate its lightweight processing capability.

We chose Jester dataset (Materzynska et al., 2019) for our experiments. Jester is a dataset of video clip gesture recognition collected using an ordinary camera, consisting of 27 types of hand gestures recorded by 1,376 participants in unconstrained environments, including different rooms, rotating fans, and moving animals. To the best of our knowledge, this is the largest video clip dataset showing human gestures, with 1,48,092 short video clips, each lasting for 3 s. We split the dataset into training/validation/test sets with a ratio of 80%/10%/10%. Many of the actions in this dataset are symmetric, such as “move finger to the left" and “move finger to the right," which require strong temporal modeling capabilities for accurate action recognition.

We conducted two versions of experiments with different image resolutions and network architectures. Version 1 focuses on low-cost processing, while version 2 prioritizes high accuracy.

Each action is represented as a sequence of multi-frame RGB images. For each frame, the image is resized to 112 × 112 for version 1 and 224 × 224 for version 2. We take 16 frames (T = 16) and perform simple data augmentation before inputting them into the network. The input data format for the network is [B, 16, 3, 112, 112] for version 1 and [B, 16, 3, 224, 224] for version 2.

**Figure 9:**
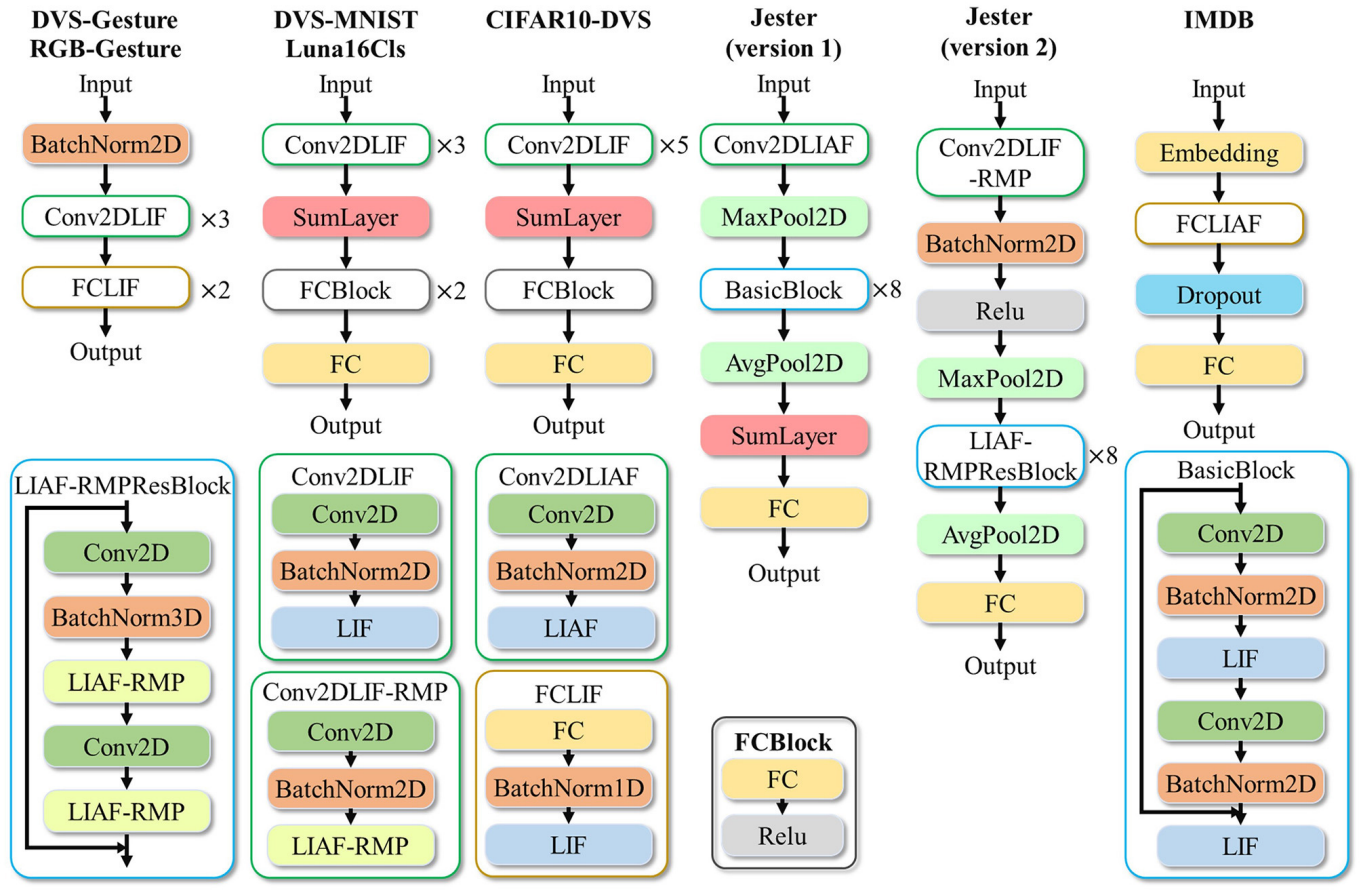
Designed neural network structures for the proposed experiments.

The neural network architecture follows a structure similar to ResNet-18, with LIAF used as the neural module for temporal processing (Figure 9). In version 1, the results of all timesteps are summed at the SumLayer and then divided by *T* for temporal dimension aggregation. The classified output is obtained through the fully connected (FC) layer. In version 2, we refine the network by substituting LIAF with the electrical coupling LIAF-RMP neural model (Wu et al., 2022b). Although the LIAF-RMP model achieves better accuracy, it incurs higher computational cost and a larger parameter size (the electrical synapses lead to a network weight size increase from 12 × 10^6^ to 24 × 10^6^).

We trained the network on the training set using a learning rate of 1e-1, weight decay of 1e-4, and momentum stochastic gradient descent (SGD) optimizer with a momentum value of 0.9. The training process utilized a cosine annealing learning rate tuning strategy. After training for 200 epochs, the top-1 classification accuracy reached 93.7% and 95.0% on the validation set for version 1 and version 2, respectively.

**Moving trajectory processing (RGB-gesture):** In some cases, we are only interested in the trajectory of the target and do not focus on static image features. In such situations, we can use the differential frame sequence as input, resulting in a simpler network. In this study, we propose the RGB-gesture dataset for this purpose. The dataset is collected using an ordinary camera and contains 10 moving gestures captured for each person similar to the DVS128 Gesture dataset (Amir et al., 2017). The video is decoded into frame data at a frequency of 25 frames per second and stored. The RGB-gesture dataset includes 760 training samples and 102 validation samples.

The RGB frames are first converted to grayscale images. We obtain a differential image by subtracting the corresponding pixel values of adjacent frames. If the pixel value changes are below a threshold, they are considered as background. Significant changes indicate moving objects and are marked as foreground. The differential results in two image channels: enhancement and weakening. After preprocessing, each sample in RGB-gesture has a dimension of [*B*, 60, 2, 40, 40], with *T* = 60.

The model is trained using the Adam optimizer with a learning rate of 1e-3 and weight decay of 1e-4. We also use the pre-trained model trained on the DVS128 Gesture dataset. The model is trained for 50 epochs, achieving a top-1 classification accuracy of 97.7% on the validation set.

#### 5.1.2. DVS signal processing

DVS is a silicon retina device that mimics the human retina's perception mechanism to perform information acquisition. The data preprocessing for converting event flow to spatiotemporal (ST) tensor is as follows: A sliding window is used to slide along the time, and an event set contains the timestamp range of events within the sliding window. Then, all events in an event set are extended into a three-dimensional vector called a frame based on their coordinate and polarity information. The positive/negative polarity events are filled in a *H* × *W* matrix according to their coordinate information in the positive/negative polarity channel, while the unfilled coordinates are set to zero. After *T* timesteps, an ST tensor with *T* frames can be obtained.

**DVS classification (CIFAR10-DVS):** CIFAR10-DVS (Li et al., 2017) is a dataset derived from the CIFAR10 dataset and collected using the DVS. We follow the event-to-ST tensor conversion methods described above. The ST tensor has a shape of [*B*, 10, 2, 128, 128], where *T* = 10, and the temporal sliding window is 5 ms. The proposed network contains five Conv2DLIF layers, followed by a SumLayer for time aggregation, and two FcBlock layers. We use the Adam optimizer with a learning rate of 1e-2 and weight decay of 1e-4 to train this network. The network's neuron parameters are set to all-share mode. The model is trained for 100 epochs, achieving a top-1 classification accuracy of 68.2% on the validation set.

**DVS recognition (DVS128 gesture):** The DVS128 Gesture dataset (Amir et al., 2017) is recorded directly from real-world scenes using a DVS camera. The DVS128 Gesture dataset has a raw spatial pixel resolution of 128 × 128. We chose a subsampled resolution of 40 × 40 (1/3.2) to save memory. To use the network structure for training, we generated event frames of size 40 × 40 by accumulating spike sequences within each 25 ms. Then, each frame was expanded into two channels depending on whether the brightness of each pixel was weakened or strengthened. Finally, multiple adjacent event frames were stacked in chronological order to obtain samples of shape [*B*, 60, 2, 40, 40]. This network structure includes three Conv2DLIF modules and two FCLIF modules. The neuron parameters were trained in the all-share mode. We used the Adam optimizer with a learning rate of 1e-2 and weight decay of 1e-4 to train this network and employed a learning rate tuning strategy during training. The network was trained for 100 epochs, achieving a top-1 classification accuracy of 94.6% (ASM) and 95.1% (CSM) on the validation set.

#### 5.1.3. 3D medical imaging

The Luna16Cls dataset is derived from the Lung Nodule Analysis 2016 (LUNA16) dataset (Setio et al., 2017). It contains CT images of 888 patients and 1, 186 nodule labels (malignant and benign) annotated by radiologists.

The preprocessing steps for the Luna16Cls dataset are as follows: (1) convert all raw data to Hounsfield Units (HU); (2) mask extraction; (3) convex hull and dilation processing; (4) gray normalization: linearly transform HU values [–1,200, 600] to grayscale values within the range of 0 to 255; and (5) downsample the dataset to a 32 × 32 image resolution to obtain samples with shape [8, 1, 32, 32]. A total of 3,795 samples are processed, with 3,416 samples used for training and the remaining samples for validation.

The Luna16Cls classification network consists of three Conv2DLIF blocks. After that, a temporal average layer is used to aggregate information along the time dimension. The model ends with an FcBlock, containing three fully connected layers for classification.

We use an SGD optimizer with a learning rate of 0.03, weight decay of 1e-4, and momentum of 0.9 to train the model on the training set. The learning rate is fine-tuned during the training process. The neural parameters are set to all-share mode. The model is trained for 20 epochs, achieving a top-1 classification accuracy of 90.4% on the validation set.

#### 5.1.4. NLP task

To test the capability of the proposed method on long sequence signal processing, such as text, a simple natural language processing (NLP) task was conducted using the IMDB dataset (Maas et al., 2011). The dataset contains 50,000 highly polarized reviews from the Internet Movie Database (IMDb). Each word in the sample data was converted into a numeric representation using a dictionary of size 1,000. Each sample data was padded to a size of 500 timesteps, resulting in a dimension of [*B*, 500] for each sample. Binary classification was used for labeling, with 0 representing negative sentiment and 1 representing positive sentiment.

The data are embedded in a tensor with shape [*B*, 500, 256], followed by an FCLIAF layer, and finally, classification is performed using a fully connected layer. This model does not have a time aggregation layer and only outputs the result of the last timestep.

For training, the Adam optimizer with a learning rate of 1e-3 and weight decay of 1e-4 is used. The learning rate is adjusted based on the epoch during training. The model is trained for 50 epochs, achieving a classification accuracy of 82.9% on the validation set.

### 5.2. Experiment results analysis

We chose these examples since they include multiple modalities, different network structures (ResNet-LIF and sequential LIF), iteration modes (IIM and EIM), neural parameter sharing mode (CSM and ASM), and various application domains, which are listed in Table 2. We also evaluated the streaming processing speed (with batch size = 1) vs. the accuracy performance of these models. It can be revealed that all the applications can work in real time both in Nvidia GPU V100 and Nvidia Jetson Xavier with streaming processing capability. Jester version 2 achieves better performance than version 1 while it suffers more computational cost. The two DVS128 Gesture implementations show that CSM achieves slightly better performance than ASM.

**Table 2:** List of the experiments.

**Source**	**Dataset**	**Network**	**Iter. mode**	**Computations (× 10^9^ Ops)**	**Weights (× 10^6^)**	**Accuracy (%)**	**GPU V100 speed (fps)**	**Xavier speed (fps)**	**GPU V100 power (W)**	**Xavier power (W)**
Video	Jester (ver. 1)	ResNet18-LIAF	EIM	ADD: 7.8 MUL: 7.8	11	93.7± 0.1	133± 5	65± 4	26.6 ± 5.9	9.4 ± 2.6
Video	Jester (ver. 2)	ResNet18- LIAF-RMP	IIM	ADD: 37.07 MUL: 37.07	24	95.0 ± 0.1	88 ± 5	47 ± 1	29.7 ± 6	18 ± 3
Video	RGB-Gesture	ConvLIAF	EIM	ADD: 1.0 MUL: 1.0	2	97.7 ± 0.1	538 ± 90	270 ± 3	34.8 ± 3.3	7.7 ± 1.4
DVS	DVS128 Gesture	ConvLIF	EIM	ADD: 1.0 MUL: 1.0	2	94.6 ± 0.6	508 ± 75	274 ± 2	34.8 ± 3.7	7.7 ± 1.4
DVS	DVS128 Gesture	ConvLIF+CSM	EIM	ADD: 1.0 MUL: 1.0	2	95.1 ± 0.8	558 ± 22	258 ± 6	36.1 ± 2.8	7.8 ± 1.1
DVS	CIFAR10-DVS	ConvLIAF	EIM	ADD: 0.84 MUL: 0.84	2.6	68.2 ± 0.5	389 ± 65	192 ± 1	35.4 ± 2.9	7.6 ± 1.3
3D Img.	Luna16Cls	ConvLIAF	EIM	ADD: 0.089 MUL: 0.089	1.2	90.4 ± 0.2	620 ± 91	267 ± 4	34.5 ± 3.5	7.4 ± 0.9
Text	IMDB	FCLIAF	EIM	ADD: 0.0024 MUL: 0.0024	0.0051	82.9 ± 0.1	2140 ± 290	1055 ± 22	34.9 ± 3.7	6.5 ± 0.3

We evaluated implementations in BIDL with different applications, network structures (We use ASM by default), neural models, iteration modes, and platforms. It shows that all these implementations achieve real-time processing on GPU servers and embedded platforms with streaming processing settings (batchsize = 1). Note that frames per second (fps) is equal to timesteps per second.

We further compared the accuracy and the computational complexity of the proposed implementations with other related work, and the comparison is revealed in Table 3. For the Jester dataset, our implementation (version 1) achieves lightweight processing which consumes half of the computations compared to the LSTM approach and similar computation to the lightweight CNN approach AdaFuse (Meng et al., 2021). Higher accuracy can also be achieved via Jester version 2, which has comparable performance to other high accuracy approaches. For DVS128 Gesture, we also achieve a better balance of performance and computational cost. For CIFAR10-DVS, we achieve better accuracy than most of the other approaches while reducing the computation by more than 4 times. For Luna16Cls, we achieve the smallest cost while maintaining accuracy. For IMDB, the proposed approach consumes only 7% of the computation compared to LSTM while maintaining accuracy (with less than 3% performance loss). In conclusion, the proposed framework can process various spatiotemporal signals with guaranteed performance and better computational efficiency.

**Table 3:** Comparison of proposed solutions with other approaches in terms of accuracy and computational costs.

**Proposals**	**Network**	**Computations (× 10^9^ Ops)**	**Accuracy (%)**
**Video processing (Jester)**			
Meng et al. (2021)	LSTM	ADD: 14.7 MUL: 14.7	93.5
Meng et al. (2021)	AdaFuse (TSN+ResNet18)	ADD: 7.6 MUL: 7.6	93.7
Jiang et al. (2019)	STM (ResNet50)	-	96.7
Zhang et al. (2020b)	STSNN (Optical flow+RGB)	-	95.7
Zhang et al. (2020a)	PAN (TSM+ResNet101)	ADD: 503.4 MUL: 503.4	97.4
This study (version 1)	ResNet18-LIAF	ADD: 7.8 MUL: 7.8	93.7
This study (version 2)	ResNet18-LIAF-RMP	ADD: 37.07 MUL: 37.07	95.0
**Video processing (RGB-Gesture)**			
This study	ConvLIAF	ADD: 1.0 MUL: 1.0	97.7
**DVS signal processing (DVS128 Gesture)**			
Massa et al. (2020)	SNN converted from CNN on Loihi	-	89.6
Amir et al. (2017)	CNN on TrueNorth	-	94.6
Kugele et al. (2020)	SNN converted from ANN	-	95.6
Khoei et al. (2020)	Converted CNN	-	95.1
Wang et al. (2019)	PointNet++	-	95.3
Bi et al. (2020)	Residual graph CNN+Res.3D	ADD: 14 MUL: 14	97.2
Wu et al. (2021)	ConvLIF	ADD: 6.8 MUL: 0.013	94.1
Wu et al. (2021)	ConvLIAF	ADD: 6.8 MUL: 6.8	97.6
This study	ConvLIF	ADD: 1.0 MUL: 1.0	94.6
This study	ConvLIF+CSM	ADD: 1.0 MUL: 1.0	95.1
**DVS signal processing (CIFAR10-DVS)**			
Cannici et al. (2019)	Attention Mechanisms	-	44.1
Sironi et al. (2018)	HATS	-	52.4
Wu et al. (2019)	iterative LIF + NeuNorm	ADD: 8.1 MUL: 8.1 ^*^	60.5
Wu et al. (2021)	ConvLIF	ADD: 3.8 MUL: 0.21	63.5
Wu et al. (2021)	ConvLIAF	ADD: 3.8 MUL: 3.3	70.4
This study	ConvLIAF	ADD: 0.84 MUL: 0.84	68.2
**3D medical imaging processing (Luna16Cls)**			
Yan et al. (2017)	Vanilla 3D CNN	-	87.3
Shen et al. (2017)	Multi-crop CNN	-	87.4
Zhu et al. (2018)	Deep 3D DPN	ADD: 26 MUL: 26 ^*^	87.1
Dey et al. (2018)	DenseNet	ADD: 0.14 MUL: 0.14 ^*^	88.4
Dey et al. (2018)	MoDenseNet	ADD: 0.14 MUL: 0.14 ^*^	90.4
Shi et al. (2020)	LIF-classification Net	-	94.1
This study	ConvLIAF	ADD: 0.089 MUL: 0.089	90.4
**Text processing (IMDB)**			
This study	LSTM	ADD: 0.4 MUL: 0.4	85.7
This study	FCLIAF	ADD: 0.0024 MUL: 0.0024	82.9

^*^ Indicates that we calculate the data based on the network structure of the corresponding study.

### 5.3. Global-local co-learning

Recent studies have shown that global-local co-learning is more resistant to noise (Wu et al., 2022a). In this study, we conducted an experiment to demonstrate the application of co-learning on a spatiotemporal network to achieve improved noise resistance performance.

To introduce noise into the data, we added background noise to the preprocessed frames of the DVS128 Gesture validation set. First, we defined a noise ratio α ∈ [0, 1]. For each validation sample, we randomly selected *n* = α × *W* × *H* pixels on each channel of every frame and set their values to 1, thereby introducing background noise. The training set remained unchanged. The network used for co-learning is similar to the one described in Section 3.2.2 and Section 5.1.2.

The experiment followed the procedure outlined below. First, we trained the network using BPTT, as described in Section 3.2.1. For comparison purposes, we performed inference on this network directly without local training on the noisy validation set, varying the value of α, and recorded the corresponding inference accuracies.

**Figure 10:**
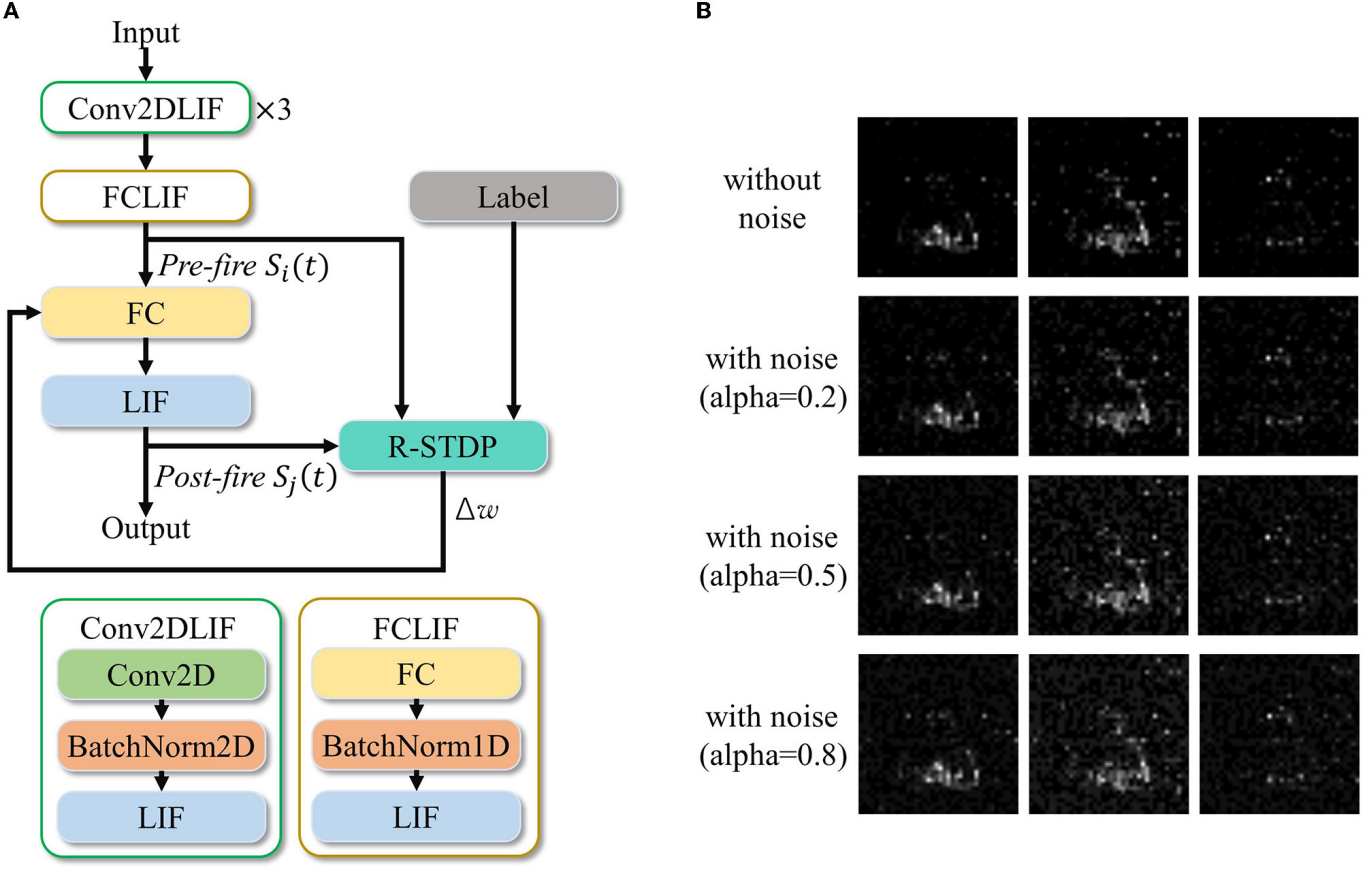
An example implementation of the BPTT and R-STDP co-learning for improved anti-noise performance. **(A)** Network architecture with R-STDP finetuning. **(B)** Visualization of the three ST frames with different noise ratios.

Next, we employed a local learning method to further refine the trained network. We adjusted the weights *W* of the last FCLIF layer using R-STDP. In the figure, *W* is represented by a 256x11 matrix, and we used seven neurons to represent each category, resulting in a total of 11 categories. The weight update process was streamed, with each sample being updated at every timestep and a batch size of one. The specific architecture of the R-STDP local learning is depicted in Figure 10A, and the noisy frames are visualized in Figure 10B.

The R-STDP method we propose consists of the following three steps:

(1) Reward calculation: Calculate the mean spikes of neurons belonging to each category as $O_{i}=\frac{1}{N}\overset{N-1}{\underset{i=0}{\sum }}O_{ij}$, where *i* ∈ [0, 10] represents the 11 categories, and *N* = 7 indicates the seven neurons in each category (i.e., population coding). Then, calculate the reward *r*_*ij*_ as the difference between the label and the output spikes: *r*_*ij*_ = *L* − *O*_*i*_, where *L* denotes the label.

(2) Weight update based on R-STDP: Calculate Δ*W* using the input spike *S*_*in*_ and output spike *S*_*out*_ as follows:


(12) $$\begin{array}{l} \text{Δ}W=t_{in}\cdot S_{out}-S_{in}\cdot t_{out}. \end{array}$$

Here, the traces *t*_*in*_ and *t*_*out*_ are derived from spikes and are updated as


(13) $$\begin{array}{l} t_{in/out}=\text{Θ}\cdot t_{in/out}+\eta \cdot S_{in/out}. \end{array}$$

(3) Update weights: *w* = *w* + *lr*·Δ*w*.

We set *lr* = 0.001, Θ = 0.95 and η = 1.0.

**Figure 11:**
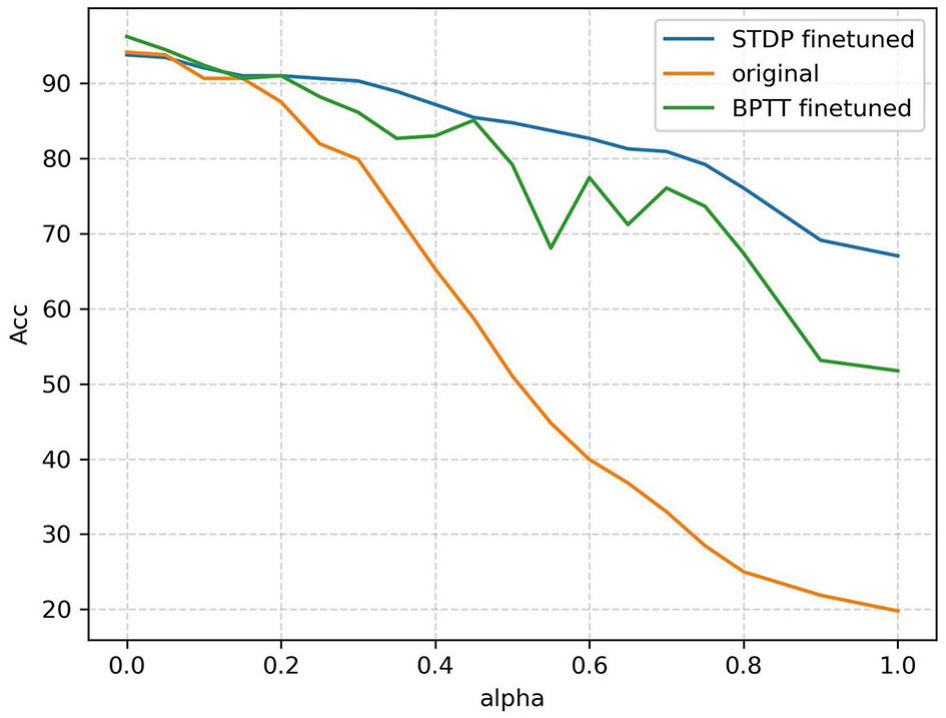
Accuracy versus noisy ratio (alpha) curves of the three approaches: BPTT without population coding (original), BPTT with population coding (BPTT finetuned), BPTT+STDP with population coding (STDP finetuned). When population coding is introduced, seven neurons represent one classification category; otherwise, one neuron represents a category.

After the training, we evaluated the accuracy of the noisy validation set. The accuracies obtained by the two methods are plotted in Figure 11. The three approaches compared are BPTT training without fine-tuning and 11 output neurons (original curve); BPTT with R-STDP fine-tuning and 77 output neurons (STDP fine-tuned curve); and BPTT with BPTT fine-tuning and 77 output neurons (BPTT fine-tuned). The results demonstrate that in a noisy environment, global-local co-learning achieves better anti-noise performance compared to a pure BPTT-trained network with the same number of output neurons and significantly outperforms the non-population coding BPTT version.

### 5.4. LIF+ neural models

To verify the performance impact of various neural models, we re-implemented CIFAR10-DVS using several LIF+ configurations while keeping all other settings the same as illustrated in Section 3.1.1. We tested a subset of configurations and present the results in Table 4. While some neural dynamics are not compatible with gradient backpropagation and result in lower performance, certain configurations still achieve similar results to the default LIF model. This indicates the admissibility of exploring neural models for spatiotemporal tasks. Future studies can focus on identifying neural models with improved performance.

**Table 4:** LIF+ performance with selected configurations on the CIFAR10-DVS dataset, where the “All default” configuration is equivalent to a LIF model, and other configurations are defined in Section 3.1.1.

**Configuration**	**Accuracy (%)**
All default (A=0, B=0, C=0, D=0, E=0)	67.86
A=0, B=3, C=0, D=0, E=0	67.43
A=0, B=3, C=1, D=0, E=0	67.63
A=0, B=3, C=2, D=0, E=0	67.56

## 6. Discussions

### 6.1. Single frame (image) processing

Processing images within this framework is possible by encoding the image into a sequence of specialized frames using a coding scheme, such as binary frames for SNN processing. However, we assume that SNN's multiple timestep processing may require more computation compared to single-frame processing in CNNs. Since most of the computation in CNNs is attributed to the convolutional operation, which does not benefit significantly from sparse (event-driven) processing, the computational cost of an SNN for processing a frame is unlikely to be significantly lower than an equivalent-sized CNN. Therefore, our framework does not aim to process image sources.

### 6.2. Neural coding

For the entire network, spikes are emitted based on neural dynamics and trained using gradient-based or local algorithms. Thus, no dedicated neural coding is assigned to any neuron, and the neurons fire based on their dynamics. Regarding input data, it is possible to represent temporal-free data using neural coding in the temporal domain. For instance, a pixel in the input image can be represented by a spike train with a corresponding firing rate (rate coding) or first firing time (temporal coding). The network's output in BIDL is primarily in analog format, but it can be encoded into a spike train.

### 6.3. Direct training vs. conversion methods

In this framework, we utilize direct training for DSNN. Conversely, there are approaches (Tran et al., 2015; Xingjian et al., 2015) that convert artificial neural networks (ANNs) to SNNs, achieving high accuracy. However, these conversion methods are primarily designed for image processing without temporal domain information in the source data. The temporal domain is generated using neural coding during the conversion stage. Our study focuses on spatiotemporal processing, where the temporal information already exists in the source data. Additionally, direct training achieves better performance and enables fine-tuning, offering more flexibility compared to post-training conversion methods.

### 6.4. Strengths and limitations of BIDL

Prior studies (Chen and Gong, 2022; Chen et al., 2022) have also proposed spatiotemporal investigations aiming to establish brain-inspired models and verify visual processing functions with biological evidence. In comparison, BIDL focuses more on solving real-world spatiotemporal tasks with DSNNs. It explores the utilization of brain-inspired technologies for spatiotemporal applications, emphasizing computational efficiency and real-time processing. Unlike some DSNN works (Wu et al., 2018; Shen et al., 2022), BIDL incorporates multiple modalities such as video and 3D imaging data, flexible neuron models, and global-local co-learning. However, BIDL has limitations in modeling sparse brain networks, especially with synaptic delays, as the computation is performed in a dense tensor format. Furthermore, it does not gain significant benefits from event-driven processing due to tensor-based convolution/linear operations. Nevertheless, BIDL achieves better efficiency through neural dynamics, which serve as lightweight processors of temporal information compared to Conv3D and ConvLSTM.

## 7. Conclusion

This study introduces a brain-inspired deep learning framework, BIDL, which provides a foundation and design flow for rapidly developing spatiotemporal applications, particularly lightweight real-time video clip analysis and dynamic vision sensor (DVS) applications. BIDL also serves as a research platform for investigating neuron models, synaptic plasticity, global-local co-learning, and network structure. Networks designed using BIDL can be easily deployed on GPU platforms and neuromorphic chips. We hope that BIDL will inspire further research in the design, exploration, and application development of bio-inspired neural networks.

## Data availability statement

The original contributions presented in the study are included in the article/supplementary material, further inquiries can be directed to the corresponding authors.

## Author contributions

ZW: conceptualization, methodology, investigation, and writing. YS: software development and investigation. JZ: software development, investigation, and data curation. HLia: writing and conceptualization. RZ: software development and methodology. HLi: software development (neuromorphic mapping). JX: review and editing. XZ: investigation (jester). YC: project administration, conceptualization, and supervision. All authors contributed to the article and approved the submitted version.

## Footnotes

^1^The commercial version of BIDL with Lynchip support is named LYNBIDL.
